# Real-world experience with pasireotide-LAR in resistant acromegaly: a single center 1-year observation

**DOI:** 10.1007/s11102-021-01185-w

**Published:** 2021-09-08

**Authors:** Maria Stelmachowska-Banaś, Izabella Czajka-Oraniec, Agnieszka Tomasik, Wojciech Zgliczyński

**Affiliations:** Department of Endocrinology, The Centre of Postgraduate Medical Education, Warsaw, Poland

**Keywords:** Acromegaly, Pasireotide-LAR, Growth hormone, Insulin-like growth factor-1, Diabetes mellitus

## Abstract

**Context:**

Pasireotide-LAR, a second-generation somatostatin receptor ligand (SRL), is recommended for patients with acromegaly as second-line treatment. Its efficacy and safety were assessed in clinical trials; however, the real-world evidence is still scarce.

**Objective:**

The aim of this study was to evaluate the impact of 1-year treatment with pasireotide-LAR on disease control and glucose metabolism in acromegaly patients resistant to first-generation SRLs.

**Design:**

A single-center prospective study.

**Methods:**

Twenty-eight patients with active acromegaly or acrogigantism on first-generation SRLs following ineffective pituitary surgery were switched to treatment with pasireotide-LAR 40 or 60 mg i.m. every 28 days. To assess the efficacy of the treatment GH and IGF-1 levels were measured every 3 months. Safety of treatment was carefully evaluated, especially its impact on glucose metabolism.

**Results:**

Complete biochemical control (GH ≤ 1 ng/mL and IGF-1 ≤ 1 × ULN) was achieved in 26.9% of patients and partial + complete response (GH ≤ 2.5 ng/mL and IGF-1 ≤ 1.3 × ULN) in 50.0% of patients. Mean GH level decrease was the largest within first 6 months (P = 0.0001) and mean IGF-1 level decreased rapidly within the first 3 months (P < 0.0001) and they remained reduced during the study. Blood glucose and HbA1c levels increased significantly within 3 months (P = 0.0001) and stayed on stable level thereafter. Otherwise, the treatment was well tolerated and clinical improvement was noticed in majority of patients.

**Conclusions:**

This real-life study confirmed good effectiveness of pasireotide-LAR in patients resistant to first-generation SRLs. Pasireotide-LAR was overall safe and well tolerated, however significant glucose metabolism worsening was noted.

## Background

Growth hormone (GH)-secreting pituitary adenoma is a leading cause of acromegaly and pituitary gigantism. The GH hypersecretion results in overproduction of insulin-like growth factor 1 (IGF-1), which contributes to somatic overgrowth, distorted body proportion (mainly involving the face and extremities) and systemic manifestations, such as upper airways obstruction, cardiovascular complications, and metabolic disorders [1–3]. The most frequent (up to 50% of patients) metabolic disorders in acromegaly resulting from direct anti-insulin effects of GH is insulin resistance leading to glucose intolerance and diabetes mellitus (DM) [4, 5]. The prevalence of glucose metabolism abnormalities in acromegaly varies considerably across different studies, but the prevalence of DM is estimated at 16–56% [6]; additionally, a positive correlation between baseline IGF-1 level and DM was found [5, 7].

Treatment of acromegaly is aimed at excising the disease-causing lesion and reducing GH and IGF-1 levels to normal values. Achieving a disease control results in normal-life expectancy and improvement in comorbidities. Transsphenoidal selective adenomectomy is considered to be a first-line treatment with reported biochemical cure rates varying between 32 and 85% depending on neurosurgeon experience, tumor size and cavernous sinus invasion. In patients with non-radical surgery, in whom re-operation is not recommended, first-generation somatostatin receptor ligands (SRLs), octreotide-LAR and lanreotide autogel are reasonable treatment options [1, 3, 8], but the efficacy is moderate (IGF-1 normalization is achieved in about 30% of patients) [9].

Pasireotide long-acting release (pasireotide-LAR) is a second-line treatment, recommended for patients with resistance to first-generation SRLs. Pasireotide-LAR is a long-acting somatostatin multireceptor ligand, with 40- and 106-fold higher binding affinity to SSTR5 than octreotide-LAR and lanreotide autogel, respectively [10, 11]. The efficacy and safety of pasireotide-LAR was assessed in a phase III clinical trial in patients with acromegaly uncontrolled on first-generation SRLs. Patients in pasireotide-LAR group achieved better biochemical disease control than patients receiving octreotide-LAR. Safety profile was similar to other SRLs, except for a higher frequency and severity of hyperglycemia and DM [12]. However, there is still not too many real-world evidence of pasireotide-LAR effectiveness and safety available in the literature.

## Aim

The aim of this study was to evaluate the impact of 1-year treatment with pasireotide-LAR on biochemical disease control and on glucose metabolism in patients with acromegaly resistant to first-generation SRLs in clinical practice.

## Methods

### Study design and patient population

In this single-center prospective study 28 adult patients (13 females, 15 males), with active acromegaly or acrogigantism on first-generation SRLs following ineffective pituitary surgery were switched to monotherapy with pasireotide-LAR between October 2018 and December 2019 and treated for 12 months. The resistance to treatment with first-generation SRLs was defined as not achieving biochemical acromegaly control (GH > 2.5 ng/mL and IGF-1 > upper limit of normal [ULN]) despite at least 6-month octreotide-LAR or lanreotide autogel therapy at maximal doses, i.e. 30 or 40 and 120 mg every 28 days, respectively. In 5 (17.9%) patients a dopamine agonist (in 2 patients cabergoline, in 3 patients bromocriptine) was added to first-generation SRL long before switching to pasireotide LAR, but did not improve acromegaly control. The patients included in the study were not suitable for re-operation. In one patient with McCune-Albright syndrome the primary treatment with surgery was not possible due to severe fibrous dysplasia of the skull. Adjuvant radiotherapy was applied in 3 (11.5%) patients, but in all cases the time elapsed since irradiation was more than 3 years. Patients with poorly controlled DM (glycosylated hemoglobin [HbA1c] > 8%) and those with visual field defect due to optic chiasm compression were excluded from the study.

The initial dose of pasireotide-LAR was 40 mg i.m. every 28 days. If patient did not achieve biochemical control (GH < 2.5 ng/mL and IGF-1 ≤ ULN for age and sex) at month 3, the dose could have been up-titrated to 60 mg i.m. every 28 days. The dose might be temporarily or permanently decreased to 20 mg if adverse reactions or over-response to treatment (IGF-1 < lower limit of normal) occurred.

If the patient’s GH did not decrease at least 50% or below 2.5 ng/mL or IGF-1 level did not decrease at least 40% comparing to the baseline after 6 months of treatment with the dose of 60 mg, the patient could be withdrawn from the study.

This study was conducted in accordance with the Declaration of Helsinki. Written informed consent was obtained from all patients for use of clinical data in research. The investigators adhered to good clinical practice guidelines. Investigations, including genetic testing, followed international guidelines and the treatment was conducted according to the therapeutic program approved by Ministry of Health.

### Assessment and assays

Patient visits were focused on acromegaly symptoms and possible side effects of treatment, physical examination (including blood pressure and heart rate), glucose metabolism evaluation and pasireotide-LAR administration were performed monthly. On each visit patients were asked to describe if the following acromegaly symptoms: fatigue, headaches, joint pains, sweating, paresthesia were unchanged, improved, deteriorated or subsided comparing to baseline. Complete biochemical and hormonal measurements were conducted at month 3, 6, 9 and 12. GH and IGF-1 concentrations were measured as biochemical acromegaly control parameters prior to the planned administration of the next pasireotide-LAR dose on the same visit. The blood samples for GH and IGF-1 were taken in the morning (9:00 to 10:00 a.m.) from fasting patients and analyzed locally using the LIAISON^®^ XL analyzer (DiaSorin, Italy) based on chemiluminescent method. The GH assay has a sensitivity of 0.05 ng/mL, an intra-assay coefficient of variation (CV) of 1.93% for a GH concentration of 1.18 ng/mL and inter-assay CV of. 3.77% for a GH concentration of 1.11 ng/mL. The intra-assay CV is 4.59% for an IGF-1 concentration of 189.3 ng/mL and inter-assay CV is 4.3% for an IGF-1 concentration of 202.6 ng/mL.

Hyperglycemia monitoring and management were based on the American Diabetes Association and European Association for the Study of Diabetes [13]. As part of the monitoring, patients were required to self-monitor their fasting blood glucose at specified intervals (e.g. once a week for the first three months), and, in addition, fasting plasma glucose was collected at each study visit and HbA1c level was checked every 3 months. Antidiabetic medications were permitted and could be initiated or adjusted at investigators’ discretion for the management of hyperglycemia during the study. Additionally, liver function parameters: alanine transaminase (ALT), aspartate transaminase (AST) and bilirubin levels were measured every 3 months. Gadolinium-enhanced pituitary MRI (magnetic resonance imaging) and abdomen ultrasound were performed for each patient at baseline, after 6 and 12 months of treatment. On MRI tumor size was measured and optic chiasm was assessed. A pituitary tumor volume change of ≥ 20% from screening was considered significant [1]. Abdomen ultrasound was primarily aimed at detecting cholelithiasis. For cardiac safety reasons, each patient had electrocardiograms (ECGs) done and corrected QT intervals (QTc) were calculated at baseline and monthly for the first 3 months following start of treatment and dose augmentation.

Patients with confirmed familial isolated pituitary adenoma (FIPA) (at least 2 members of family with a history of pituitary adenoma) were tested for *AIP* mutation courtesy of prof. Marta Korbonits as previously described [14].

### Endpoints

The key efficacy endpoint was the proportion of patients achieving complete biochemical control, i.e. GH ≤ 1 ng/mL and age- and sex-adjusted normal IGF-1 (IGF-1 ≤ 1 × ULN) [15]. Additional endpoints were the proportion of patients with partial + complete response defined as achieving GH ≤ 2.5 ng/mL and IGF-1 ≤ 1.3 × ULN and overall changes in GH and IGF-1 levels. For safety assessment fasting plasma glucose and HbA1c, ALT, AST and bilirubin levels changes from baseline were also assessed.

### Statistical analyses

Data were presented as mean and standard deviation (SD) or median and interquartile range (IQR). The t-Student test was used for normally distributed quantitative variables, and Wilcoxon signed-rank test for non-normally distributed data. The strength of the association between variables was measured with use of Pearson's correlation coefficient. One-way ANOVA test was used to compare the means of more than two groups. The P-values < 0.05 were considered statistically significant. All statistical analyses were performed using R software (version 3.6.3).

## Results

### Patient population

In total, 26 patients (12 females, 14 males), out of 28 patients enrolled in the study, were included in the final analysis. Two patients were excluded prematurely (after 3 months of treatment) from the study: one patient due to poor DM control aggravated by de novo endogenous hypercortisolemia and one patient due to lack of compliance with the study visits. None of the patients was excluded from the 12-month follow-up due to lack of efficacy. Mean age of patients was 42.6 (SD 12.8, range 23–67) years. Twelve patients (46.2%) were 40 years old or younger including 6 (23.1%) patients of age ≤ 30 years (Table 1). There were 4 patients with FIPA and 2 patients with McCune-Albright syndrome, diagnosed on the basis of clinical symptoms with histopathological confirmation of fibrous dysplasia of the bone. Patients with FIPA were tested for *AIP* mutations as previously described [14], and in all the results were negative. Altogether 5 patients within the studied group had acrogigantism (height above 2 standard deviations for Polish population that is 191.4 cm for males and 176.9 cm for females) [16], among them one patient with McCune-Albright syndrome and one with FIPA.

**Table 1:** Baseline characteristics

Characteristic	Pasireotide-LAR, n = 26
Age	
Mean (SD), year	42.6 (12.8)
≤ 30 years old, n (%)	6 (23.1)
≤ 40 years old, n (%)	12 (46.2)
Female, n (%)	12 (46.2)
Previous treatment, n (%)	
Surgery	25 (96.0)
Radiotherapy	3 (11.5)
Octreotide-LAR	16 (61.5)
Lanreotide autogel	10 (38.5)
Metabolic status, n (%)	
DM	4 (15.4)
Prediabetes	19 (73.1)
Normal	3 (11.5)
Diabetes medications, n (%)	
Metformin	20 (76.9)
Metformin + insulin	2 (7.7)
Metformin + GLP-1 analogue	1 (3.8)
IGF-1, ng/mL	
Mean (SD)	573.9 (174.1)
IGF-1, ULN	
Mean (SD)	2.3 (0.7)
GH, ng/mL	
Mean (SD)	3.9 (2.9)
Fasting plasma glucose, mg/dL	
Mean (SD)	111.0 (21.3)
HbA1c, %	
Mean (SD)	5.9 (0.4)
ALT, U/L	
Mean (SD)	20.3 (7.9)
AST, U/L	
mean (SD)	20.9 (6.5)
Bilirubin, mg/dL	
Mean (SD)	0.7 (0.3)

Prior to study enrollment all patients underwent debulking pituitary surgery, except for a patient with McCune-Albright syndrome. All included patients were resistant either to octreotide-LAR (n = 16, 61.5% patients) or to lanreotide autogel (n = 10, 38.5%) used at maximal doses.

At baseline mean IGF-1 level was 2.3 × ULN (age- and sex-specific) (573.9 ng/mL) and mean GH concentration was 3.9 ng/mL.

Baseline mean fasting plasma glucose level was 111.0 mg/dL, and mean HbA1c was 5.9%. Prior to study enrollment 4 (15.4%) and 19 (73.1%) patients were diagnosed with DM and prediabetes, respectively. Thus, the majority of study patients had anti-diabetic treatment at baseline, including 20 (76.9%) patients taking metformin, 2 (7.7%) patients treated with metformin and insulin and 1 (3.8%) patient with metformin and GLP-1 (glucagon-like peptide-1) analogue.

Pasireotide-LAR dose was increased after 3 months to 60 mg in 21 (80.8%) patients due to incomplete biochemical response. In one patient who normalized IGF-1 level after 3 months, the dose was decreased to 20 mg due to worsening of DM control.

### Efficacy

**Fig. 1:**
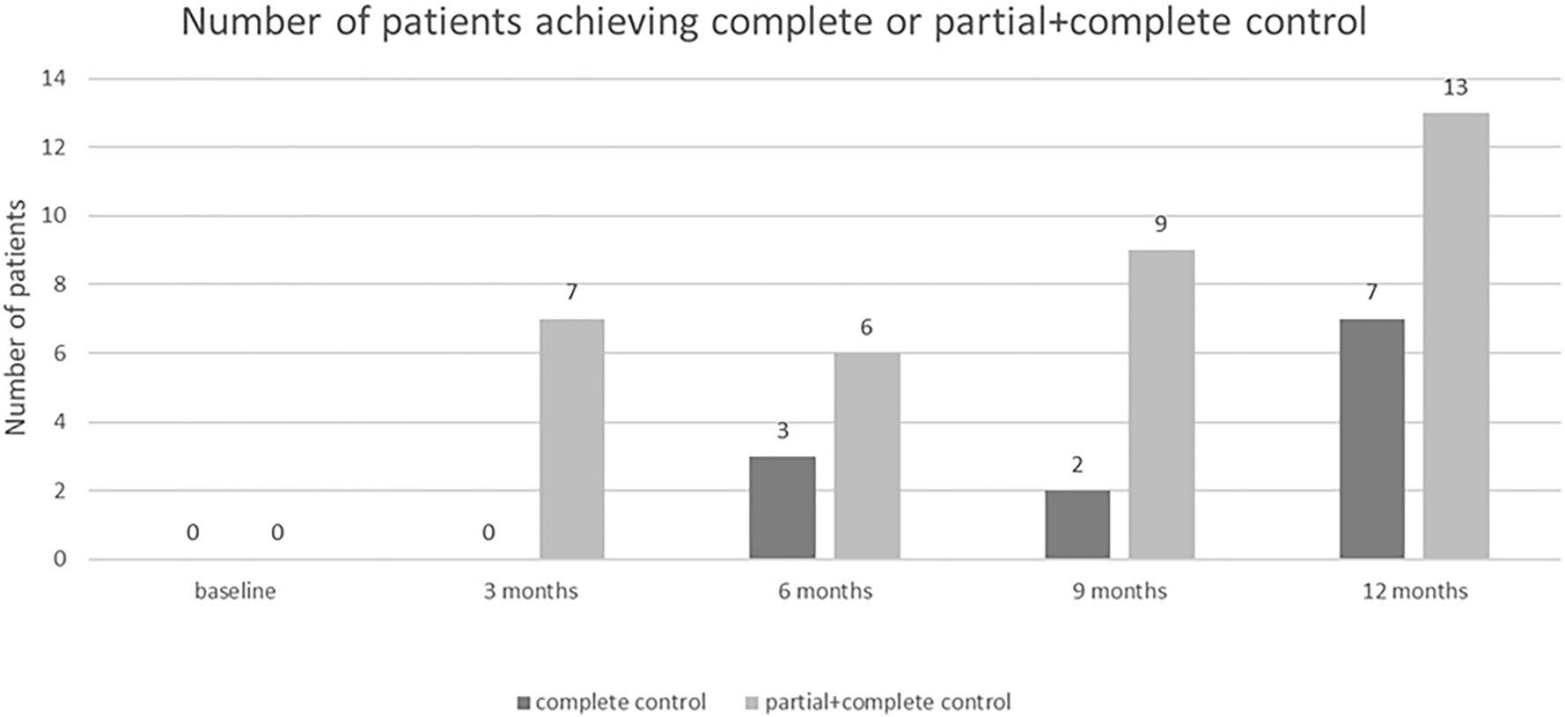
Number of patients achieving complete or partial + complete control over time

GH level ≤ 1 and ≤ 2.5 ng/mL was achieved after 1-year treatment period by 11 (42.3%) and 21 (80.8%) patients, respectively. Ten patients (38.5%) achieved normal IGF-1 level (IGF-1 ≤ 1 × ULN). IGF-1 ≤ 1.3 × ULN was observed in 14 (53.8%) of patients. Complete control (GH ≤ 1 ng/mL and IGF-1 ≤ 1 × ULN) was achieved after 1 year in 7 (26.9%) patients (4 males, 3 females; mean age 48.1 years) and partial + complete response (GH ≤ 2.5 ng/mL and IGF-1 ≤ 1.3 × ULN) in 13 (50.0%) patients (5 males, 8 females; mean age 44.0 years) (Table 2, Fig. 1). Among patients achieving complete response there were 1 (25.0%) patient with FIPA (receiving 60 mg), 2 (100.0%) patients with McCune-Albright syndrome (receiving 40 mg) and 3 (60.0%) patients with acrogigantism (2 patients receiving 40 mg and 1 patient on 60 mg). In those patients with FIPA or with acrogigantism who did not normalize biochemical parameters there was a significant decrease in mean IGF-1 level after 12-month treatment comparing to baseline: − 194.44 ng/mL (95% CI: − 294.56; − 94.32), P = 0.006 and − 215.6 ng/mL (95% CI: − 372.90; − 58.30), P < 0.05, respectively.

**Table 2:** Proportion of patients achieving complete or partial + complete biochemical control and with IGF-1 ≤ 1 × ULN, IGF-1 ≤ 1.3 × ULN, GH ≤ 1 ng/mL or GH ≤ 2.5 ng/mL at month 12

Parameter	Follow-up	n (%)
GH		
GH ≤ 1 ng/mL	Baseline	1 (3.8)
	12 Months	11 (42.3)
GH ≤ 2.5 ng/mL	Baseline	11 (42.3)
	12 Months	21 (80.8)
IGF-1		
IGF-1 ≤ 1 × ULN	Baseline	0 (0.0)
	12 Months	10 (38.5)
IGF-1 ≤ 1.3 × ULN	Baseline	1 (3.8)
	12 Months	14 (53.8)
GH and IGF-1		
Complete control	Baseline	0 (0.0)
	12 Months	7 (26.9)
Partial + complete control	Baseline	0 (0.0)
	12 Months	13 (50.0)

**Fig. 2:**
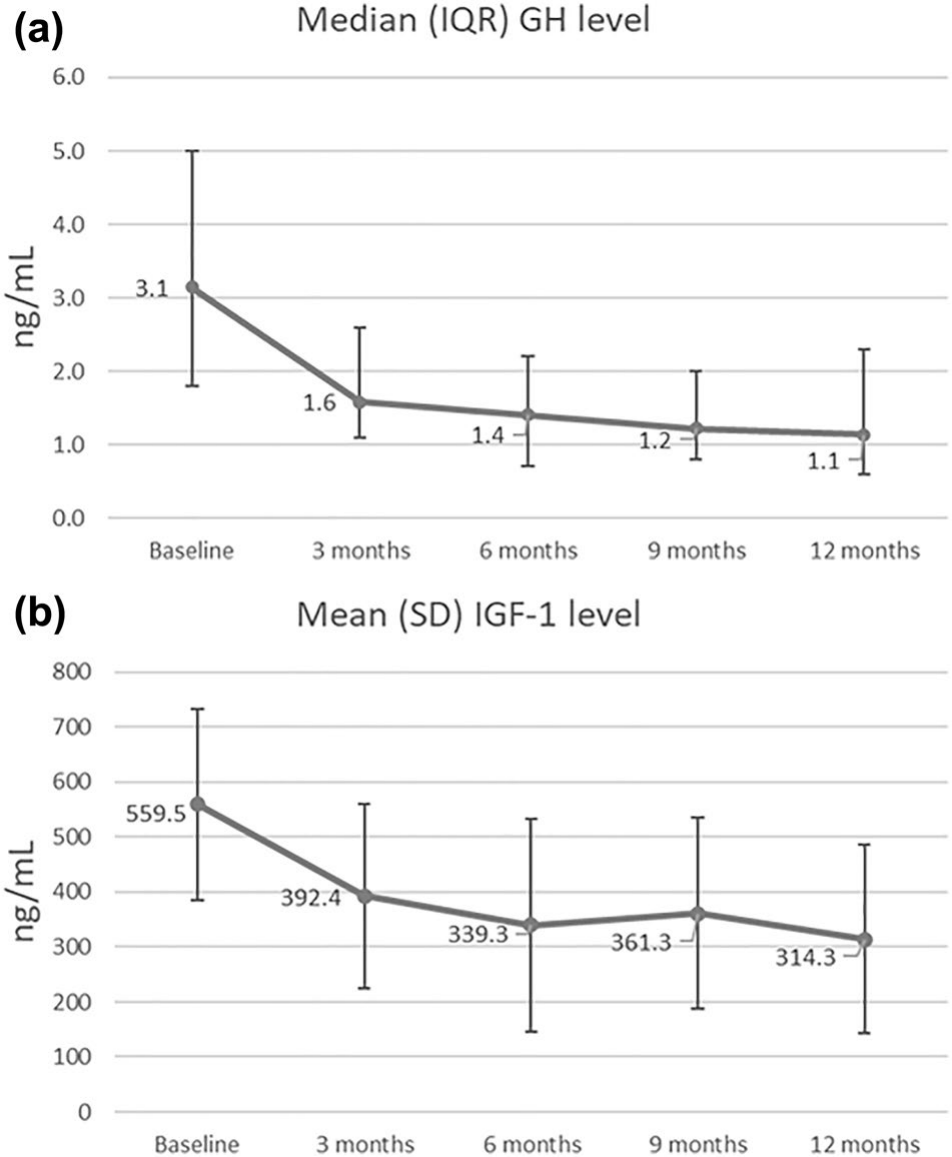
Median GH (**A**) and mean IGF-1 (**B**) concentrations at each checkpoint during 12-month observation

The magnitude of mean GH level decrease was the largest within first 6 months (mean change from baseline: − 1.67 ng/mL, 95% CI: − 2.43, − 0.91, P = 0.0001). At 9-month visit, a slight increase of GH level was observed, but then it decreased again during the last 3 months of observation. Mean IGF-1 level decreased rapidly within the first 3 months (mean change from baseline: − 163.64 ng/mL, 95% CI: − 209.16, − 118.12, P < 0.0001) and remained low during 12-month follow-up. However, a slight increase at month 9 was also noticed (Table 3, Fig. 2A, B).

**Table 3:** Median GH and mean IGF-1 concentrations and mean changes from baseline of GH and IGF-1 concentrations at each checkpoint during 12-month observation

Follow-up	Median (IQR)	Change from baseline	
		Median (95% CI)	P-value
GH [ng/mL]			
Baseline	3.1 (1.8, 5.0)	n/a	n/a
3 Months	1.6 (1.1, 2.6)	− 0.95 (− 1.69, − 0.47)	< 0.0001
6 Months	1.4 (0.7, 2.2)	− 1.18 (− 1.49, − 0.85)	< 0.0001
9 Months	1.2 (0.8, 2.0)	− 1.44 (− 1.97, − 0.95)	< 0.0001
12 Months	1.1 (0.6, 2.3)	− 1.16 (− 1.67, − 0.89)	< 0.0001

Follow-up	Mean (SD)	Change from baseline	
		Mean (95% CI)	P-value
IGF-1 [ng/mL]			
Baseline	573.9 (174.1)	n/a	n/a
3 Months	413.3 (167.9)	− 163.64 (− 209.16, − 118.12)	< 0.0001
6 Months	371.1 (194.1)	− 202.75 (− 253.87, − 151.62)	< 0.0001
9 Months	390.9 (174.2)	− 182.99 (− 222.81, − 143.17)	< 0.0001
12 Months	336.8 (171.3)	− 237.10 (− 272.73, − 201.47)	< 0.0001

**Fig. 3:**
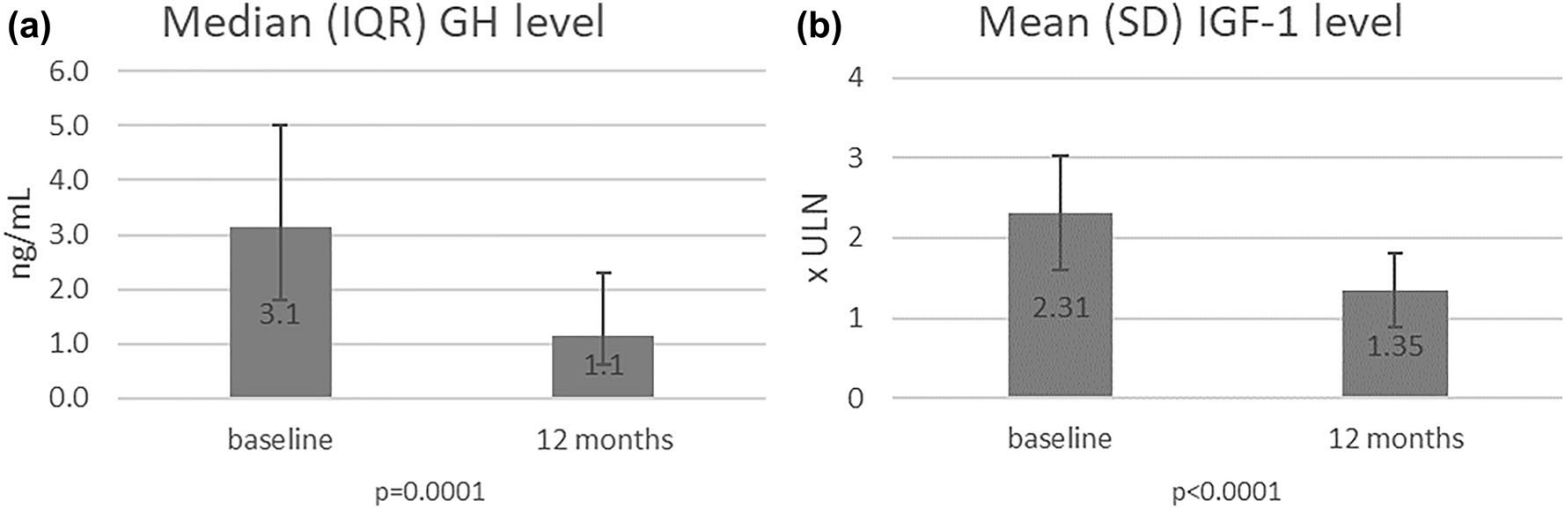
Median GH (**A**) and mean IGF-1 level expressed as a multiple of the upper limit of the normal (× ULN) (**B**) at baseline and after 12 months of pasireotide-LAR treatment

Similar to GH level, mean IGF-1 level expressed as a multiple of the upper limit of the normal (× ULN) was significantly lower after 12 months of pasireotide LAR treatment (P < 0.0001) (Fig. 3A, B).

The increase in IGF-1 level at month 9 was observed in 18 (69.2%) patients, and in 17 of them this increase was greater than 5% (the value determined as the minimum difference at which the change for the entire sample is statistically significant).

In the subgroup of patients treated with pasireotide-LAR in a dose increased to 60 mg after 3 months of study, mean IGF-1 change was similar as in patients treated with 20/40 mg. The largest decrease of IGF-1 level was observed during first 3 months of treatment, when patients actually were receiving a dose of 40 mg per month (Table 4).

**Table 4:** Mean IGF-1 change from last visit, in patients receiving 20/40 and 60 mg every 4 week

Follow-up	Mean IGF-1 change (SD) [ng/mL]		
	Pts receiving 20/40 mg (n = 5)	Pts receiving 60 mg (n = 21)	P-value
0–3 month	− 152.25 (89.58)	− 165.81 (115.59)	0.7791
3–6 month	− 49.15 (43.37)	− 30.70 (98.28)	0.5232
6–9 month	28.58 (15.74)	18.25 (90.15)	0.6221
9–12 month	− 25.73 (23.14)	− 63.63 (44.64)	0.0080

An improvement in the signs and symptoms of acromegaly (headache, fatigue, perspiration, osteoarthralgia, and paresthesia) from baseline was observed throughout the study even in patients who did not achieve complete biochemical control. Among patients with a shift in symptom severity, all patients had a shift to less severe symptoms. Among patients with headaches at baseline (n = 18), in 11 patients (61.1%) headaches resolved and in 3 improved (16.7%). Fatigue improved in 20/26 patients (76.9%), osteoarthralgia in 11/21 patients (52.4%), excessive sweating in 10/14 patients (71.4%) and paresthesia in 11/15 patients (73.3%).

### Glucose metabolism parameters

Pasireotide-LAR treatment resulted in significant increase of mean fasting plasma glucose level. The largest change was observed in first 3 months, and it remained quite stable for the next 9 months, achieving mean 125.2 (SD 23.8) mg/dL in month 12 vs. mean 111.0 (SD 21.3) mg/dL at baseline. HbA1c concentration also increased significantly during first 3 months and stayed on similar level (mean for month 12: 6.5 (SD 0.6) % vs. 5.9 (SD 0.4) % at baseline) (Table 5).

**Table 5:** Mean fasting glucose level and HbA1c level during 12-month treatment

Follow-up	Mean (SD)	Change from baseline	
		Mean (95% CI)	P-value
Fasting plasma glucose [mg/dL]			
Baseline	111.0 (21.3)	n/a	n/a
3 Months	124.1 (24.4)	13.06 (4.65, 21.47)	0.0037
6 Months	121.9 (19.6)	10.84 (3.24, 18.44)	0.0070
9 Months	126.3 (22.9)	14.97 (7.32, 22.62)	0.0005
12 Months	125.2 (23.8)	14.99 (8.55, 21.42)	0.0001
HbA1c [%]			
Baseline	5.9 (0.4)	n/a	n/a
3 Months	6.3 (0.6)	0.42 (0.26, 0.59)	< 0.0001
6 Months	6.3 (0.7)	0.39 (0.22, 0.56)	0.0001
9 Months	6.4 (0.6)	0.49 (0.33, 0.64)	< 0.0001
12 Months	6.5 (0.6)	0.53 (0.38, 0.68)	< 0.0001

*n/a* not applicable

At 12 months, 11 (42.3%) patients were diabetic, 15 (57.7%) patients were prediabetic, and 1 (3.8%) patient had normal metabolic status, while at baseline 4 (15.4%), 19 (73.1%) and 3 (11.5%), respectively. Six (23.1%) patients had their anti-diabetic treatment intensified, by adding a new drug, i.e. sulfonylurea or DPP-4 (dipeptidyl peptidase 4) inhibitor, and most of them had their metformin increased to maximum tolerated dose.

There was a tendency for greater mean HbA1c increase from baseline in a subgroup receiving the dose of 60 mg than in a subgroup on pasireotide-LAR 20/40 mg, but the difference did not reach statistical significance. However, pasireotide-LAR dose was not increased to 60 mg in only 5 patients, so the sample size of 20/40 mg subgroup is relatively low; therefore, the calculations bias may arise.

The comparison of outcomes achieved by patients > 40 and ≤ 40 years old shows a statistically significant difference. Mean HbA1c change from baseline was significantly higher in older patients at each checkpoint (Table 6).

**Table 6:** Mean HbA1c change from baseline during 12-month follow up in patients < 40 and ≥ 40 years old

Follow-up	Mean HbA1c change from baseline [%]		SD (95% CI)	P-value
	> 40 years old (n = 14)	≤ 40 years old (n = 12)		
3 months	0.66	0.14	0.52 (0.28, 0.77)	0.0002
6 months	0.62	0.12	0.50 (0.22, 0.78)	0.0011
9 months	0.64	0.32	0.32 (0.04, 0.60)	0.0288
12 months	0.76	0.27	0.49 (0.28, 0.71)	0.0001

The results of Pearson correlation analysis demonstrated that the level of HbA1c showed a positive correlation with patients age (coefficient: 0.4188, P = 0.0332 for 12 months), i.e. older patients are statistically more likely to have increased (higher) HgbA1c level (with the exception of 9 months outcome).

Baseline metabolic status seems not to be a prognostic factor of HbA1c level changes. The comparison of diabetic vs. the rest of patients, as well as comparison of diabetic and prediabetic patients vs. normal metabolic status patients, show no significant differences (P-value after 12 months 0.3750 and 0.1482, respectively). Most of the patients (23, 88.5%) had anti-diabetic treatment at baseline. In these patients similar HbA1c level changes were observed as in patients without any anti-diabetic treatment (P = 0.3213 after 12 months). However, achieving reliable results may be difficult due to low number of patients in subgroups and the intensification of antidiabetic treatment.

**Fig. 4:**
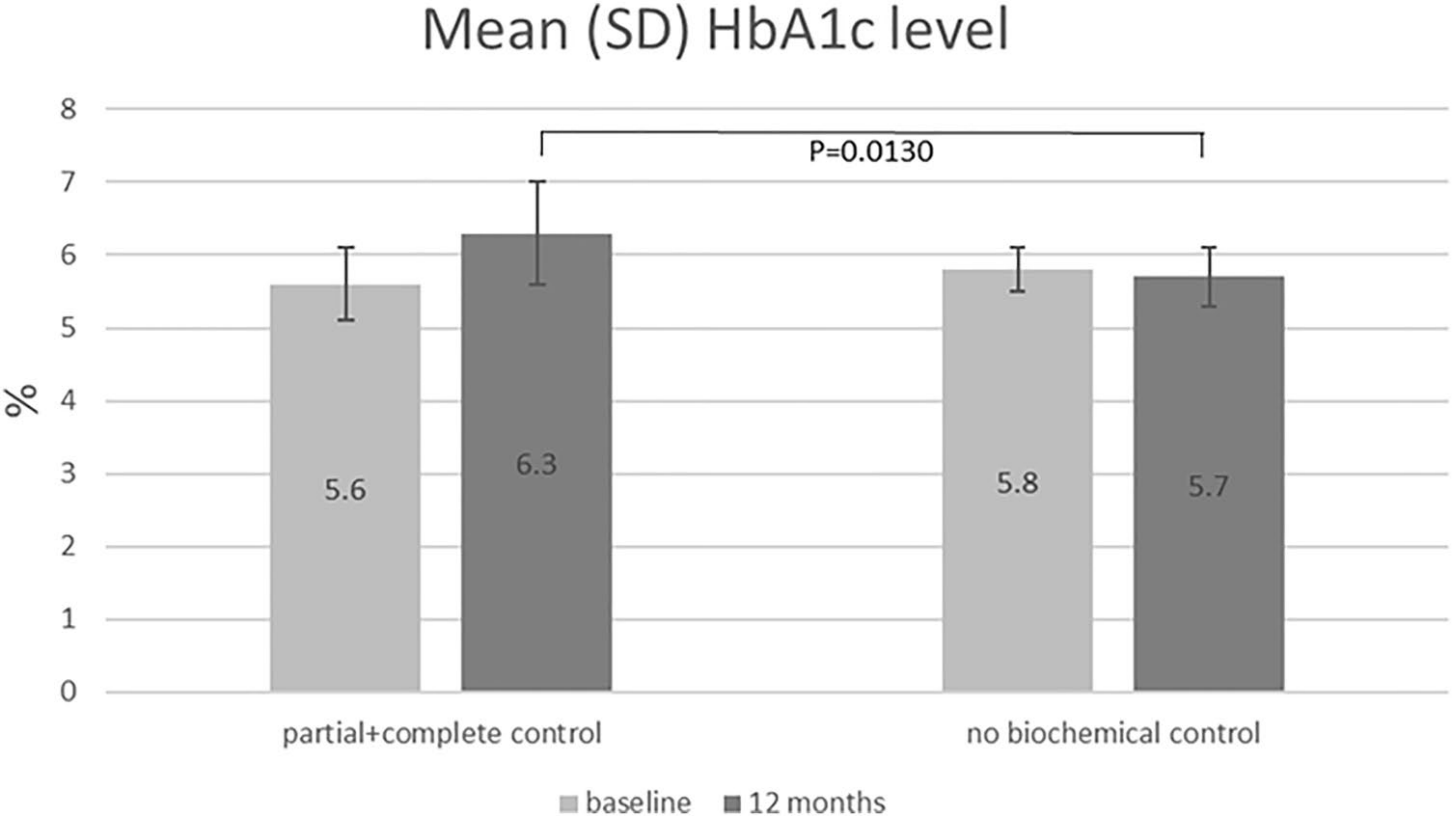
Mean HbA1c level in patients with and without a biochemical control after 12-month treatment

Two of 7 (28.6%) patients with complete control and 1 of 6 (16.7%) patients with partial control were diabetic at baseline and after 12-month of pasireotide-LAR treatment. Mean HbA1c level change after 12 months of treatment was 0.7% in patients with partial + complete response and − 0.1% for the rest of patients (uncontrolled). The difference between subgroups was statistically significant at month 12 (P = 0.0130) (Fig. 4). In patients, for whom diabetes treatment was intensified during the study (one or more anti-diabetic drugs were added), similar HbA1c level changes were observed as in the rest of patients (P = 0.0631).

Five (19%) patients had HbA1c level ≥ 7% but ≤ 7.5% after 12 months of pasireotide-LAR treatment.

Treatment with pasireotide-LAR was generally well tolerated. In addition to hyperglycemia, the most common adverse effects were gastrointestinal symptoms i.e., abdominal pain, flatulence and diarrhea which occurred in 10 patients (38.5%). Hair loss was reported in 2 patients (7.7%). Bradycardia was observed in 1 patient (resolved after beta-blocker discontinuation), but no case of QTc interval prolongation ≥ 450 ms was found. In one patient cholelithiasis was diagnosed on ultrasound (treated with first-generation SRL for 3 years before study enrollement), and in one patient with a normal abdominal ultrasound at baseline the examination showed gallbladder sludge. In one patient symptomatic bile duct obstruction due to gallstones occurred requiring endoscopic retrograde cholangiopancreatography. In one patient with cholelithiasis before study enrolment acute cholecystitis occurred requiring surgery. ALT, AST and bilirubin levels were assessed every 3 months, and no significant changes were observed.

MRI images showed a significant remnant tumor shrinkage in 2 patients. All other patients had stable size of tumor remnants and no patient experienced optic chiasm compression leading to visual field defects during treatment. In one patient with McCune-Albright syndrome due to pituitary hyperplasia, a decrease in pituitary volume was observed during treatment with pasireotide-LAR.

## Discussion

The primary therapeutic goal of acromegaly treatment is achieving biochemical control and tumor shrinkage, as well as relieving symptoms. Currently available treatment options include SRLs, dopamine agonists, and growth hormone receptor antagonist. Octreotide-LAR and lanreotide autogel, first-generation SRLs, are well-tolerated, but their effectiveness in terms of normalization of IGF-1 levels is only about 30% [9]. In the case of treatment failure, pasireotide-LAR could be used, with the effectiveness in the treatment of acromegaly resistant to first-generation SRLs demonstrated in numerous clinical studies [12, 17–20]. All of them showed a significant decrease in GH and IGF-1 levels during pasireotide-LAR treatment compared to first generation SRLs in a higher proportion of patients. However, currently there are only limited amount of relevant real-world evidence.

The results of our observational study were consistent with those obtained from clinical trials, or they even can be a proof of higher effectiveness, as the older criterium for biochemical acromegaly control (GH < 2.5 ug/mL and IGF-1 < 1 × ULN) was used in other trials [12, 21]. Complete biochemical control, defined as GH ≤ 1 ng/mL and IGF-1 ≤ 1 × ULN, was achieved by 7 (26.9%) patients and complete + partial response (GH ≤ 2.5 ng/mL and IGF-1 ≤ 1.3 × ULN) was reported in 13 (50.0%) patients, with uncontrolled disease during prolonged octreotide-LAR or lanreotide autogel treatment before switching to pasireotide-LAR. After 1-year treatment both mean GH and mean IGF-1 level decreased significantly compared to baseline. The response to treatment was noticeable early. The greatest decrease in IGF-1 level was observed after 3 months and in GH level after 6 months of treatment. Therefore, results of our study confirm the effectiveness of pasireotide-LAR in daily clinical practice.

Almost half (46.2%) of the patients in this study were aged 40 or younger. It should be noted that younger patients, especially males (who constituted the majority of studied group), are more often resistant to treatment with SRLs [22]. A similar relationship was demonstrated for patients with higher GH and IGF-1 levels at diagnosis [23]. Patients with FIPA, McCune-Albright syndrome and acrogigantism accounted for a large portion of the studied population. It is known that these patients more commonly present with resistant acromegaly. Until now pegvisomant in monotherapy or in combination with first generation SRLs, rather that pasireotide-LAR, has been specifically mentioned in the context of these subgroups treatment [24–27]. In our study substantial proportion (55%) of those patients showed a positive response to pasireotide-LAR treatment. One of 4 patients with FIPA, both patients with McCune-Albright syndrome and 3 out of 5 patients with acrogigantism achieved complete biochemical control. However, in all of them biochemical and clinical improvement were observed (significant IGF-1 change after 12 months: P = 0.0057 for patients with acrogigantism and P = 0.0223 for patients with FIPA). There are limited data in the literature on the efficacy of pasireotide-LAR treatment in patients with FIPA, both with or without germline *AIP* mutation [28, 29]. In all 4 patients with FIPA the results of germline *AIP* mutation test were negative. Although in this subgroup only one patient achieved complete acromegaly control after one year of treatment, other patients achieved significant reduction in GH and IGF-1 levels. It should be also emphasized that even if pasireotide-LAR treatment did not result in GH and IGF-1 levels normalization, a significant alleviation of acromegaly-related symptoms such as headache, impaired functioning, fatigue, excessive sweating, joint pain, and paresthesia, was observed. Thus, pasireotide-LAR could be a valuable alternative to pegvisomant or combined pegvisomant and first generation SLRs therapy for management of patients with possible genetic background of acromegaly or acrogigantism, resistant to first-generation SRLs, e.g. patients with FIPA or McCune-Albright syndrome. Pasireotide-LAR monotherapy is more cost-effective compared to combination treatment and it also has advantage over pegvisomant when there is tumor growth concern [30]. In our group no one had growth of the remnant tumor mostly located in one or both cavernous sinuses during pasireotide-LAR therapy, including patients with FIPA, acrogigantism or McCune-Albright syndrome. Three patients had significant decrease of the tumor residue, including one patient with McCune-Albright syndrome, who did not have primary surgery.

In all patients, the highest GH and IGF-1 levels reduction was observed in the first 6 or 3 months of treatment, respectively. Moreover, as these parameters were assessed every 3 months, it was possible to observe transient increase in GH and IGF-1 levels, most likely due to the phenomenon of tachyphylaxis caused possibly by “down-regulation” of somatostatin receptors. It consists in the fact that after some time at pasireotide-LAR treatment the levels of GH and IGF-1 increase, and then decline again. In our study the transient worsening of outcomes was clearly noticeable in the 9th month of treatment. The increased IGF-1 concentration at this time point cannot be explained only by within-subject variability in IGF-1 levels [31, 32], as patients were included in the study at different time points (from October 2018 to December 2019).This observation requires further real-life studies on larger groups of patients to see if the problem of tachyphylaxis exists during treatment with pasireotide-LAR of acromegaly patients. So far, tachyphylaxis, i.e. a sudden decrease in pasireotide effectiveness after previous successful treatment, has only been observed in Cushing’s disease [33–35].

Despite the largest decrease in IGF-1 level during the first 3 months, as well as a temporary increase in IGF-1 after 9 months of treatment, it is important to evaluate the effectiveness after one year of treatment. The percentage of patients who obtained partial or complete biochemical control was higher after 12 months than after 3 months (complete response: 26.9 vs 0%, partial + complete response: 50.0 vs 23.1%).

A typical side effect of pasireotide-LAR treatment is hyperglycemia. Although disorders of carbohydrate metabolism are one of the symptoms of the disease itself, pasireotide can exacerbate them due to its mechanism of action. Compared to octreotide, it shows 30-, 5- and 40-fold greater affinity for sstr1, sstr3 and sstr5 receptor, respectively, and 2.5-fold lower affinity for the sstr2 receptor. Pasireotide-LAR inhibits insulin secretion because sstr2 and sstr5 receptors, to which this drug has a particularly high affinity, are present on the surface of pancreatic beta cells [6]. Another mechanism involved in diabetes development during pasireotide treatment is the inhibition of incretin hormones, such as GLP-1 and gastric inhibitory polypeptide [36, 37]. Therefore, as hyperglycemia is an expected effect of pasireotide use, blood glucose levels should be closely monitored throughout the treatment period. In the case of some disorders, therapeutic management is recommended according to general guidelines for pre-diabetes and diabetes mellitus, i.e. the implementation of an appropriate diet, physical activity, as well as antidiabetic medications, including metformin in the first line [38]. Due to the mechanism of action of pasireotide, the use of incretin drugs, i.e. DPP-4 inhibitors or GLP-1 analogues is also reasonable [30]. All patients receiving hypoglycemic drugs in our study were managed according to the guidelines. Most of the patients were diagnosed with diabetes (15.4%) or pre-diabetes (73.1%) at study enrollment, likely due to uncontrolled acromegaly. The use of pasireotide-LAR in this group of patients led to the deterioration of carbohydrate metabolism indices, therefore antidiabetic treatment was intensified (by increasing the dose of metformin or introducing another drug). At 12 months, already 42.3% patients were diabetic and 57.7% prediabetic. Nonetheless, in most patients the degree of glucose metabolism deterioration was mild and majority of them (n = 21, 80.8%) achieved targeted HbA1c level < 7% after one year of pasireotide-LAR treatment. Effective and early management of hyperglycemia in patients treated with pasireotide-LAR is very important as hyperglycemia and diabetes are long-term cardiovascular risk factors. Moreover, proactive treatment of hyperglycemia may positively influence the long-term compliance of patients with acromegaly taking pasireotide-LAR [30].

Similarly to other studies, we found significant and rapid increase in fasting glucose (111.0 to 124.1 mg/dL) and HbA1c level (5.9 to 6.3%) during 3 months after pasireotide-LAR treatment initiation with plateau thereafter [21, 39]. It could be a result of close monitoring of carbohydrate metabolism parameters and prompt intensification of antidiabetic treatment or the mechanism of action of pasireotide-LAR itself.

We noticed that HbA1c level after one-year treatment was higher in patients achieving partial or complete control (6.3%) than in patients with no biochemical control (5.7%). This is contradictor to Schmid et al. study, showing that incidence of hyperglycemia and HbA1c levels were similar in responders and non-responders to pasireotide-LAR treatment, but they were more closely related to the baseline fasting plasma glucose. Among patients with baseline FPG > 100 mg/dL, hyperglycemia was observed more frequently than in those with baseline FPG ≤ 100 mg/dL, especially if they were using higher dose of pasireotide (60 vs. 40 mg) [21]. Similar conclusions were drawn from the Sheppard et al. study, in which hyperglycemia during pasireotide-LAR treatment was reported in 19% and 45% of patients with baseline FPG < 100 mg/dL and > 100–125 mg/dL, respectively. Among patients with HbA1c levels within the reference range (< 5.7%) before pasireotide treatment, 23% had HbA1c level ≥ 6.5% at the last measurement. However, diabetes developed in 55% of patients with impaired glucose tolerance (HbA1c level between 5.7 and < 6.5%) [39].

It can be concluded, that despite achieving acromegaly control after pasireotide-LAR treatment, glycemic control worsened requiring hypoglycemic treatment intensification. That could be an evidence that better responsiveness to pasireotide-LAR therapy manifesting as better biochemical control might be linked to higher sensitivity to metabolic disturbances and increased risk of hyperglycemia.

In our study we did not see that the baseline metabolic status has been a predictive factor for development or worsening of hyperglycemia. However, our subgroup sizes could have been not sufficient for achieving reliable results, mostly due to low number of patients with normoglycemia at baseline.

Other reason for worse glycemic control among our patients achieving partial or complete response might be their age. Only 2 out of 13 patients with partial + complete control were < 40 years old. Since a positive correlation between age and risk for DM development was observed in general population, higher HbA1c concentration in patients with acromegaly biochemical control may be rather because of their age. The mean HbA1c change during one-year treatment was significantly larger in patients > 40 years old than in those ≤ 40 years old (0.76 vs. 0.27% change from baseline, P = 0.0001).

Comparison of the results between the patients receiving an increased pasireotide dose and a dose of 40 mg (or 20 mg in one case) throughout the study, showed no statistically significant differences in terms of HbA1c level. Thus, it does not appear that higher pasireotide-LAR doses increase the risk of further worsening of hyperglycemia. However, it should be remembered that the group of patients with not increased dose was small (6 patients). Therefore, further studies involving more patients are necessary to confirm this observation.

It is also worth emphasizing that the general study results could have been influenced by the results of individual patients with extremely active disease, for example one patient with a baseline GH concentration of 14.1 ng/mL Such data significantly impact the estimates; however, their inclusion results from the observational nature of the study, reflecting actual clinical practice.

In the light of the available evidence and the results of this study, it is clear that pasireotide-LAR is an effective treatment option in patients with acromegaly resistant to first-generation SRLs. Many patients, including patients with acromegaly in the course of FIPA or McCune-Albright syndrome may achieve clinically significant improvement during pasireotide-LAR treatment. However, it is important to initiate close monitoring of carbohydrate metabolism parameters and applying appropriate antidiabetic management.

## Conclusions

Real-world data for pasireotide-LAR treatment in patients with acromegaly resistant to first-generation SRLs proved that it is an effective therapeutic option. This study confirmed the effectiveness of pasireotide-LAR also in patients with genetic background of acromegaly and acrogigantism after one-year observation. The significant increase of glucose and HbA1c levels were observed, as expected based on previously published studies. The strongest increase of these parameters occurred during first 3 months and they remain stable until the end of study.

## Disclosure

MSB and WZ received lecture honoraria from Recordati and Pfizer. ICO and AT received lecture honoraria from Recordati.
